# Genomic Landscape of Multidrug Resistance and Virulence in *Enterococcus faecalis* IRMC827A from a Long-Term Patient

**DOI:** 10.3390/biology12101296

**Published:** 2023-09-29

**Authors:** J. Francis Borgio, Reem AlJindan, Lujeen H. Alghourab, Rahaf Alquwaie, Razan Aldahhan, Norah F. Alhur, Doaa M. AlEraky, Nehal Mahmoud, Noor B. Almandil, Sayed AbdulAzeez

**Affiliations:** 1 Department of Genetic Research, Institute for Research and Medical Consultations (IRMC), Imam Abdulrahman Bin Faisal University, Dammam 31441, Saudi Arabia; fbalexander@iau.edu.sa (J.F.B.); raldahhan@iau.edu.sa (R.A.); norah.f.s.2@gmail.com (N.F.A.); 2 Department of Microbiology, College of Medicine, Imam Abdulrahman Bin Faisal University, Dammam 31441, Saudi Arabia; nmhosin@iau.edu.sa; 3 Summer Research Program, Institute for Research and Medical Consultations (IRMC), Imam Abdulrahman Bin Faisal University, Dammam 31441, Saudi Arabia; 2170004781@iau.edu.sa; 4 Master Program of Biotechnology, Institute for Research and Medical Consultations (IRMC), Imam Abdulrahman Bin Faisal University, Dammam 31441, Saudi Arabia; 2230500195@iau.edu.sa; 5 Department of Biomedical Dental Science, Microbiology and Immunology Division, Collage of Dentistry, Dammam 31441, Saudi Arabia; 6 Department of Clinical Pharmacy Research, Institute for Research and Medical Consultations (IRMC), Imam Abdulrahman Bin Faisal University, Dammam 31441, Saudi Arabia; nbalmandil@iau.edu.sa

**Keywords:** *Enterococcus*, pathogen, Saudi Arabia, multidrug-resistant, mobile genetic element, genome mapping, antibiotic resistance genes, virulence, resistant mutation

## Abstract

**Simple Summary:**

A highly virulent, multidrug-resistant *Enterococcus faecalis* IRMC827A strain was found in a Saudi Arabian hospital. The strain carries antimicrobial resistance genes and mobile genetic elements, making it resistant to various antibiotics. It also carries virulence factors associated with adherence, biofilm formation, and spreading multidrug resistance. The study highlights the importance of monitoring multidrug-resistant *E. faecalis* colonization and infection in hospitalized patients, as it is a serious pathogen.

**Abstract:**

We report on a highly virulent, multidrug-resistant strain of *Enterococcus faecalis* IRMC827A that was found colonizing a long-term male patient at a tertiary hospital in Khobar, Saudi Arabia. The *E. faecalis* IRMC827A strain carries several antimicrobial drug resistance genes and harbours mobile genetic elements such as Tn6009, which is an integrative conjugative element that can transfer resistance genes between bacteria and ISS1N via an insertion sequence. Whole-genome-sequencing-based antimicrobial susceptibility testing on strains from faecal samples revealed that the isolate *E. faecalis* IRMC827A is highly resistant to a variety of antibiotics, including tetracycline, doxycycline, minocycline, dalfopristin, virginiamycin, pristinamycin, chloramphenicol, streptomycin, clindamycin, lincomycin, trimethoprim, nalidixic acid and ciprofloxacin. The isolate IRMC827A carries several virulence factors that are significantly associated with adherence, biofilm formation, sortase-assembled pili, manganese uptake, antiphagocytosis, and spreading factor of multidrug resistance. The isolate also encompasses two mutations (G2576T and G2505A) in the *23S rRNA* gene associated with linezolid resistance and three more mutations (*gyrA* p.S83Y, *gyrA* p.D759N and *parC* p.S80I) of the antimicrobial resistance phenotype. The findings through next-generation sequencing on the resistome, mobilome and virulome of the isolate in the study highlight the significance of monitoring multidrug-resistant *E. faecalis* colonization and infection in hospitalized patients. As multidrug-resistant *E. faecalis* is a serious pathogen, it is particularly difficult to treat and can cause fatal infections. It is important to have quick and accurate diagnostic tests for multidrug-resistant *E. faecalis*, to track the spread of multidrug-resistant *E. faecalis* in healthcare settings, and to improve targeted interventions to stop its spread. Further research is necessary to develop novel antibiotics and treatment strategies for multidrug-resistant *E. faecalis* infections.

## 1. Introduction

*Enterococcus faecalis* is a non-sporulating, facultatively anaerobic, and Gram-positive bacteria [1]. It is a causative agent of several pathological conditions, including surgical wound infections, infective endocarditis and central line-associated bloodstream infections [2,3]. Enterococci was first discovered by Thiercelin in 1899. However, until 1984 they were considered part of Streptococcus [4,5,6]. Contemporarily, more than 50 morphologically and biochemically diverse species belong to *Enterococcus* [1,3].

*E. faecalis* was first isolated from a patient with infective endocarditis in 1906 [7]. Moreover, *E. faecalis* has been isolated from plants, water, soil, sewage, fermented cheese, and dairy food [8]. In the last century, *E. faecalis* was one of the leading causes of hospital-acquired infections because of its multidrug resistance nature, with few therapeutic options. The dramatic increase in prevalence is due to the bacteria’s versatility in accommodating nutrition-poor environments and diverse ecological niches, such as pH, hypertonic and hypotonic conditions and temperature, as well as its ability to defeat the infection control interventions employed in hospitals [9,10,11,12,13]. *Enterococcus* is a known human intestinal flora inhabitant. Its opportunistic infections in immune-compromised individuals, along with patients receiving broad-spectrum antibiotic treatment or requiring extended hospitalization, are well studied [14,15]. Antimicrobial drugs have been the cornerstone of medical treatment during the last few decades. Since then, the infection survival rate has declined. Yet, the widespread utilization of antimicrobial drugs has been the selective pressure leading to antimicrobial resistance [16,17,18]. Despite the various applied infectious control interventions across the hospital settings, as well as the wild environments concerning antimicrobial drug use, antimicrobial resistance still continues to rise [10,19]. A comprehensive study in European countries has demonstrated that Enterococci species accounted for approximately 6.1% to 17.5% of the isolated pathogens and were associated with the highest rate of mortality [20]. Likewise, a global meta-analysis study has highlighted the acceleration of bloodstream infections associated with antimicrobial resistance in Southeast Asian and Eastern Mediterranean countries compared to the world [21]. It is specifically noted that there has been a rapid growth in the vancomycin-resistant Enterococci (VRE) rate since it was first reported in 1988 [22]. VRE is a serious dilemma as its infections are not easily treated, owing to the fact that vancomycin is a drug of last resort [17]. India has experienced an upsurge in the prevalence of VRE, estimated to have risen up to 10% since 2000 [23]. Furthermore, the latest evidence has revealed the presence of VRE in food, animals, and wild environments which holds the risk of interstrain transmission of resistance genes and might require more complicated interventions and multisectoral collaboration [17]. The expansion of antimicrobial resistance has encompassed other antimicrobial drugs, such as tigecycline, linezolid, and daptomycin, yet they are still considered reasonable options against enterococcal infections [24].

The translocation process of *E. faecalis* remains controversial. Some studies have proposed that it occurs when *E. faecalis* crosses the lymphatic system after failing to be neutralized through phagocytosis by intestinal epithelial cells, dendritic cells, or other tissue-resident leukocytes. Others have claimed it happens when a slight quantity of bacteria diffuses across the intestinal barrier into the bloodstream. [13,25,26]. However, this minor leakage is not a threat in immunocompetent individuals; the innate system is sufficient to conquer such invaders [13,27,28]. Over-colonization of bacteria is a significant risk factor for developing intestinal infection, and it is certainly associated with *E. faecalis* emergences; it competes with other commensals to colonize human intestines [27].

The spread of multidrug-resistant microorganisms is not uncommon in Saudi Arabia. In recent years, several hospitals across the kingdom have been reporting resistant bacteria. The vancomycin-resistant genes in Enterococcus species were first identified in Riyadh in 1993, and ever since, the provenance of these genes has steadily increased [29]. The following genes—*van A*, *van B*, and *van C*—were identified in the central region and are estimated to occur in approximately 3.5% to 4% of the detected isolates [30,31], while in the eastern region, the prevalence rate of vancomycin-resistant genes is higher compared to other regions of the country at 6.1% [32]. However, a genomic analysis study of *E. faecalis* in the western region has identified over 34 resistance-associated genes linked to various types of commonly used antibiotics [33].

Aggregation substance is the foremost virulence factor for pathogenesis. It permits the bacteria to adhere to and colonize the host’s epithelial tissues [34]. *E. faecalis* secretes substances that present bactericidal and cytocidal activities. In addition, virulent *E. faecalis* strains express a pore-forming exotoxin named cytolysin [35]. For instance, Cytolysin coded by *cylLL* and *cylLS* genes hemolyze the host’s cells [28,36,37,38]. Gelatinase encoded by the *gelE* gene hydrolyzes gelatin [28,38,39]. Lastly, serine protease encoded by the *sprE* gene disintegrates casein [28,38]. Conjointly, these virulence factors play a role in forming biofilm communities. Biofilm communities are cells encased in an exopolymer matrix and can adhere to biotic and abiotic surfaces, exchange genetic material, and facilitate the spread to extra-intestinal sites. Most importantly, it gives them the beneficial feature of being resistant to antibiotics and immunological responses [40,41,42].

Various studies including a recent systematic review were conducted to give prominence to the prevalence and emergence of multidrug-resistant bacteria and fungi from the Arabian Peninsula, including Saudi Arabia [43,44,45,46]. Around 80 species of bacteria and fungi were reported in a recent systematic review from the Arabian region [46]. Unfortunately, Saudi Arabia reported the highest number of multidrug-resistant bacteria among the other Arabian countries and demonstrated the highest mortality rate. *E. faecalis* accounts for 256 out of 533 cases caused by *Enterococcus* species from the Arabian Peninsula [46]. Considering the great genomic plasticity of *E. faecalis,* which allows the bacteria to disseminate the resistant genes [6,47], an increase in the incidence of *E. faecalis* after drug administration for treating COVID-19 patients was reported from the study region—the Eastern Province of Saudi [48]. A thorough genomic analysis of *E. faecalis* in the eastern region of Saudi Arabia has not been previously investigated in the literature. Hence, the objective of this study is to sequence the whole genome of *E. faecalis* IRMC827A and to perform analysis using various bioinformatics platforms. The isolate was evaluated for the presence of genes associated with multidrug-resistant virulent factors as well as phenotypic mutations.

## 2. Materials and Methods

### 2.1. Ethical Approval

Imam Abdulrahman Bin Faisal University’s ethical committee reviewed and approved this project (IRB-2022-01-398). The 1964 Helsinki Declaration and its following revisions, as well as comparable ethical principles, were followed during every procedure.

### 2.2. Isolation of Bacteria and DNA Extraction

Using cycloserine, cefoxitin, and fructose agar media and a faecal sample from a male patient who was clinically suspected of having a gastrointestinal infection and had recently experienced antibiotic exposure and diarrhoea, a pathogenic strain was isolated. Six predisposing antimicrobial agents (ciprfloxacin, gentamicin, flagyl, meropenem, vancomycin, and tazocin) have been associated with infection. The pathogenic strain was isolated using cycloserine, cefoxitin, and fructose agar media. A positive strain was cultivated on acyclo-serine cefoxitin fructose agar selective medium (CCFA) (MOLEQULE-ON, Auckland, New Zealand). The Gentra Puregene Yeast/Bact. Kit (Qiagen, Hilden, Germany) was used to extract the whole DNA. Thermo Scientific’s Nanodrop 2000 (Waltham, MA, USA) was used to evaluate the purity, quality, and amount of genomic DNA in accordance with the manufacturer’s recommendations. The isolate was PCR amplified, and the *16S rRNA* gene was sequenced (GenBank Accession No: OR533998) and analysed as we described earlier to confirm the strain as bacteria [45,49].

### 2.3. Genome Mining for Multidrug-Resistant Genes

The genome of the isolate was sequenced using an Illumina HiSeq system (Illumina, San Diego, CA, USA). Genomic DNA was sheared randomly to construct three-read libraries. The paired-end fragment libraries were sequenced according to the Illumina HiSeq system’s protocol. Raw reads of low quality from paired-end sequencing were discarded and other reads were assembled using SOAPdenovo v1.05 software. The paired readings were put together and annotated as previously described [45,50] using the RAST tool kit (RASTtk 1.3.0) [51] and PATRIC (BV-BRC 3.28.5) [52]. We determined the taxonomy of the IRMC827A’s genome and estimated the average G + C content and contig count using the predictions for the proteins and their roles in gene ontology (GO) [53], enzyme commission (EC) [54], pathways [55], subsystems of protein complexes [56] and protein family types [57]. Specific source databases for known transporters [58], antibiotic-resistant genes [59], virulence factors [60,61], and drug targets [62,63], were used for identifying speciality genes in the IRMC827A genome. Plasmid multilocus sequence typing was used for detecting known plasmid types of IRMC827A [64]. Anti-microbial resistance (AMR) genes were detected using k-mer-based methods [52]. Phylogenetic analysis was completed using 100 genes from the NCBI reference for the IRMC827A’s genome in addition to representative genomes by Mash/MinHash (Mash v2.3) [65] aligned with MUSCLE [66] and a matrix analysis with fast bootstrapping [67,68]. Metagenomic read mapping was conducted through k-mer alignment against the selected template using the VFDB (2019) and CARD (2020) databases [69].

Resistance phenotypes of IRMC827A were predicted using ResFinderFG (Version 2.0) using a functional metagenomic antibiotic resistance database. LRE-Finder (Version 1.0) was applied to detect the mutations in the *23S rRNA* gene and genes encoding linezolid resistance (*optrA*, *cfr*, *cfr(B)* and *poxtA*) in Enterococci [69,70]. Pathogenic protein families and mobile genetic elements associated with antibiotic resistance in the IRMC827A were predicted using PathogenFinder (Version 1.1) [71] and MGE [72], respectively.

## 3. Results

The collected stool sample was subjected to isolating anaerobic bacteria and the isolated bacterial strain, IRMC827A, was initially identified as *Enterococcus* IRMC827A using *16S rRNA* gene sequencing and analysis. In order to identify the genetic impact in the genome of IRMC827A, the whole genome of the strain was sequenced successfully, and annotated (Table 1).

**Figure 1:**
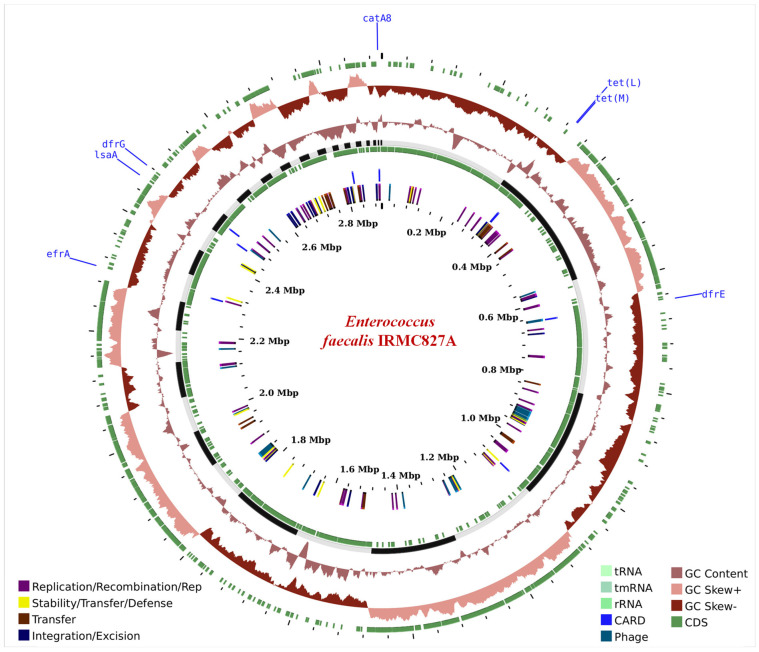
A circular graphical display of the distribution of the genome annotations in *Enterococcus faecalis* IRMC827A. This includes the antimicrobial resistance genes projected on the outer ring with names coloured blue.

**Figure 2:**
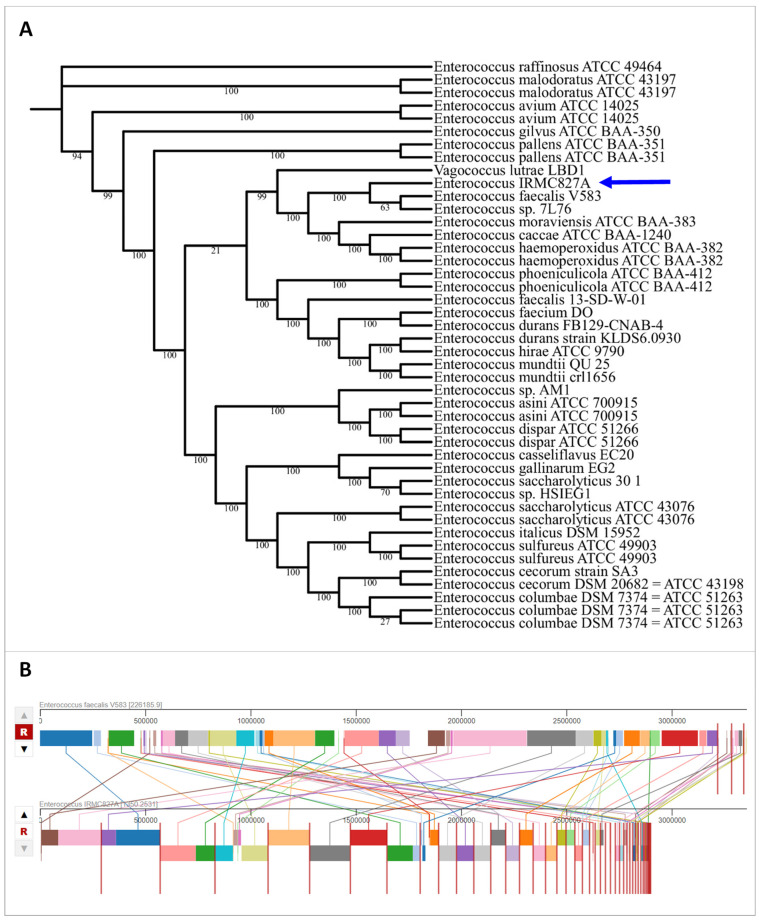
Phylogenetic tree and genome comparison of IRMC827A genome. (**A**) Phylogenetic tree of IRMC827A genome. Blue arrow indicates the genome of IRMC827A. Statistics used for phylogenetic tree analysis: alignment program—mafft; branch support method—RAxML fast bootstrapping; requested genomes and number of genomes with data—44; single-copy genes requested, found and number of protein aligned—100; max allowed deletions and duplications—0; number of aligned amino acids—39,722; protein alignment time—346.3 s; number of aligned nucleotides—119,166; number CDS alignments—100; RAxML time—8526.6 s; RAxML likelihood -2,334,654.7903; (**B**) genome comparison between *E*. *faecalis* IRMC827A and *E*. *faecalis* V583 representing homologous regions.

The IRMC827A genome (GenBank accession No: JAVLSN000000000; SubmissionID: SUB13827866; BioProject ID: PRJNA1014890) was assembled into 42 contigs with an average G+C content of 37.34% and a total length of 2,899,764 bp (Table 1). This IRMC827A genome belongs to the superkingdom Bacteria, and its taxonomy is cellular organisms > Bacteria > Terrabacteria group > Firmicutes > Bacilli > Lactobacillales > Enterococcaceae > Enterococcus > *Enterococcus faecalis.* There are 2 ribosomal RNA (rRNA) genes, 41 transfer RNA (tRNA) genes, and 2889 protein-coding sequences (CDS) in this *E. faecalis* IRMC827A genome (Table 1). Figure 1 displays a circular graphical representation of the distribution of *E. faecalis* IRMC827A genome annotations as well as a summary of the distinct biological process or structural complex for the genome. The phylogenetic tree (Figure 2), the number of speciality genes (Table 2 and Table 3), the antimicrobial resistance gene details (Table 2), the functional classification (Figure 1), and the genome comparison between *Enterococcus faecalis* IRMC827A and *Enterococcus faecalis* V583 (Figure 2) all show that the IRMC827A genome is similar to that of *Enterococcus faecalis*. In the examination of the proteins, 2250 proteins with known functions and 639 hypothetical proteins were found (Table 1). A total of 686 proteins had EC numbers and 564 had GO designations among the proteins with functional assignments. There are 2813 genus-specific protein families (PLFams) and 2848 cross-genus protein families (PGFams) in the genome of *E. faecalis* IRMC827A.

### WGS-Based Antimicrobial Susceptibility for Antimicrobial Resistance

**Figure 3:**
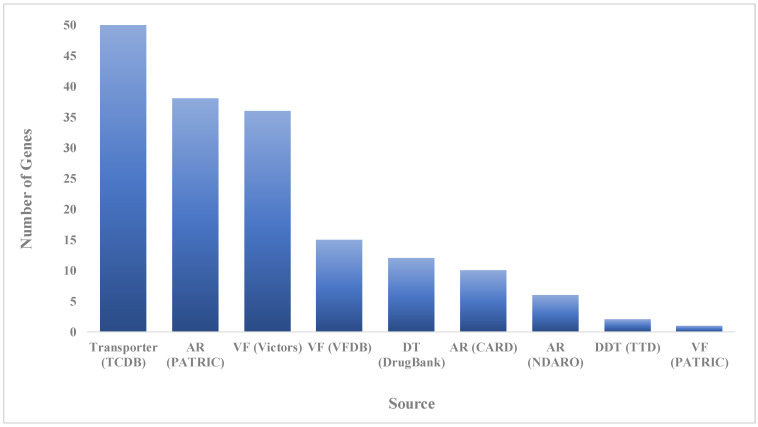
Number of speciality genes identified in the genome mining of *E. faecalis* IRMC827A and the homology identified from the specific source database. AR: antibiotic resistance; VF: virulence factor; DT: drug target; CARD: Comprehensive Antibiotic Resistance Database; NDARO: National Database of Antibiotic Resistant Organisms; PATRIC: Pathosystems Resource Integration Center; TCDB: Transporter Classification Database; VFDB: Virulence Factor Database; NDARO: National Database of Antibiotic Resistant Organisms; TTD: Therapeutic Target Database.

The CARD (Comprehensive Antibiotic Resistance Database), PATRIC (Pathosystems Resource Integration Center), NDARO (National Database of Antibiotic Resistant Organisms) databases and ResFinder were used for identifying acquired antimicrobial resistance genes using the whole genome of *E. faecalis* IRMC827A, and revealed more than 30 antimicrobial resistance genes with various antimicrobial resistance mechanisms (Table 2 and Figure 3). The *E. faecalis* IRMC827A genome has the highest number (*n* = 19) of genes connected to antibiotic targets in susceptible species. Three genes (*LiaF*, *LiaR*, and *LiaS*) involved in regulator modulating expression of antibiotic resistance genes were also identified in the IRMC827A genome. Two genes were found in each category such as antibiotic inactivation enzyme, antibiotic target protection protein and efflux pump conferring antibiotic resistance in association with the mechanism of resistance in the genome of IRMC827A.

Fifty-two virulence factors were identified in the genome of IRMC827A through ResFinder, VFDB, Victors and PATRIC (Table 3). Virulence factors are significantly associated with adherence, biofilm formation, sortase-assembled pili, manganese uptake, ABC transporter, antiphagocytosis, exoenzyme, and spreading factor of multidrug resistance in the genome of IRMC827A (Table 3 and Table S1). Metagenomic read mapping of the genome of *E. faecalis* IRMC827A against the template genomes (*Staphylococcus aureus*; *Streptococcus pneumoniae*; *Enterococcus faecium*; *E. faecalis*; *Clostridium difficile*; *Geobacillus stearothermophilus* and *E. faecalis*) using antibiotic resistance database exposed significant genes (*p* value = 1 × 10^−26^) (Table S2).

Plasmid multilocus sequence typing of IRMC827A revealed the presence of genes repA (GenBank: AB374546.1; Location: 83,003..84,010) and *HMPREF0351_12738* (GenBank: CP003584.1; Location: 24,026..25,231) in *E. faecalis* plasmid pMG2200 and *E. faecium* DO plasmid 1, respectively, based on known plasmid types with 100% identity. ResFinderFG-based analysis for identifying resistance phenotypes of IRMC827A identified a resistance phenotype to chloramphenicol (Hit name: cat; 100% identity), tetracycline (Hit name: tet_efflux; 100% identity) and cotrimoxazole (Hit name: dfr; 98.18% identity) (Figure S1) based on a functional metagenomic database. Antimicrobial-resistant phenotype analysis exposed resistance phenotype to 13 antimicrobial agents (tetracycline, doxycycline, minocycline, dalfopristin, virginiamycin m, pristinamycin iia, chloramphenicol, streptomycin, clindamycin, lincomycin, trimethoprim, nalidixic acid and ciprofloxacin) in *E. faecalis* IRMC827A (Tables S3 and S4). Tetracycline, ciprofloxacin, and chloramphenicol-resistant phenotypes specific to *E*. *faecalis* were observed in the IRMC827A genome (Table S4). Two mutations G2576T and G2505A in the *23S rRNA* gene were identified as associated with linezolid resistance; however, no mutations were detected in genes such as *optrA*, *cfr*, *cfr(B)* and *poxtA* in *E. faecalis* IRMC827A encoding linezolid resistance. There were three resistance-phenotype-associated mutations—*gyrA* p.S83Y, *gyrA* p.D759N and *parC* p.S80I—identified in the genome of *E. faecalis* IRMC827A (Table 4).

Pathogenic protein families of the IRMC827A revealed the strain as a human pathogen (probability score 0.891) with a proteome coverage of 1.97% and 55 matched pathogenic protein families (Table S5). Alignment to reference-based prediction for mobile genetic elements (MGE) associated with antibiotic resistance of the IRMC827A revealed the presence of two MGEs—integrative conjugative element (name of the MGE: Tn6009; Accession: EU399632) and insertion sequence (name of the MGE: ISS1N; Accession: M37395) (Table S6). Tn6009 (position in contig: 31342-33230) showed alignment coverage of 100% containing 1889/1889 bp with sequence identity of 99.95% and one substitution. The ISS1N (position in contig: 16162-16969) showed alignment coverage of 100% containing 808/808 bp with a sequence identity of 98.51% and 12 substitutions.

## 4. Discussion

Recent studies have found various strains of *E. faecalis* from different resources that are highly resistant to antibiotics and carry several virulence factors [3,73,74,75,76,77,78]. Due to its multidrug resistance, adaptability to nutrient-poor environments and a variety of ecological niches and limited therapy choices, *E. faecalis* was one of the main causes of hospital-acquired infections and saw dramatic increase in prevalence [9,10,11,12,13]. The presence of genes and alleles involved in antimicrobial resistance observed in the isolate IRMC827A were reported earlier from various bacterial organisms, such as multidrug-resistant *Moraxella catarrhalis* [79], a heavy-metal-resistant bacterium *Cupriavidus campinensis* S14E4C [80], *Bacillus cereus* isolated from eye shadow cosmetic products [81], *Brucella abortus* isolated from aborted fetal sheep [82], *Bacillus velezensis* CMU008 [83], multidrug-resistant *Clostridium perfringens* [45], a high-lead-resistance bacterium *Raoultella planticola* [84], a human pathogenic strain from Malaysia *Chromobacterium violaceum* [85], and multidrug-resistant *Salmonella enterica* [86], including *E. faecalis* [87].

The analysis revealed the presence of two V domain mutations such as G2576T and G2505A in the *23S rRNA* gene which are resistant to linezolid in Enterococci [69,70]. Recent studies reported an increase in the occurrence of linezolid resistance among Enterococci [77], which is in line with the observations of the current study on the linezolid resistance-associated mutations in *E. faecalis* IRMC827A. Resistance-associated mutations in various genes and matched pathogenic protein families clearly support the multidrug-resistant phenotype of *E. faecalis* IRMC827A.

One ISS1N insertion sequence, first identified in *Lactococcus lactis*, was found in the study isolate IRMC827A. These sequences are thought to be crucial for the conjugal transfer of genes involved in lactose processing between different lactic acid bacteria species. However, it is not clear in any descriptions of ISS1N mediating the transfer of virulence or resistance genes. *Weissella paramesenteroides* were found to be substantially related to IS [88,89,90]. Luna Colagrossi and colleagues reported that the insertion sequence ISS1N is an important factor for bacterial genome shaping and exogenous genetic content integration [91]. The transposase gene ISS1N was significantly abundant in microorganisms from urban wastewater compared to hospital wastewater [92]. The presence of insertion sequence ISS1N was reported in *Bacteroidetes incertae sedis*, Opitutae, and *Nitrospira* in the resistome analysis of microbial communities in river biofilms [93] and *Listeria monocytogenes* isolated from ready-to-eat foods in Chile [94]. To our knowledge, this is the first report of *E. faecalis* in Saudi Arabia that is also carrying Tn6009, along with ISS1N and other resistant genes [46]. *E. faecalis* with a mobile genetic element, the integrative conjugative element Tn6009, was reported recently in South Africa’s characteristic vancomycin-resistant phenotype [73]. Tn6009 is known to be a mobile genetic element that can transfer resistance genes between bacteria [95]. According to a Norwegian study, *E. faecalis*, which is responsible for peripheral periodontitis in hospitalised patients, has Tn916 linked with the integrase genes [96]. Another study found that Tn6009 was linked to Tn916-like components in *S. aureus* that helped spread MDR determinants that could be acquired from a variety of bacteria, with *Enterococcus* spp. having the highest rate of transmissibility [95]. The presence of transposase genes does not necessarily mean that the transposases are active. However, it is important to note that transposons can play a role in the spread of antibiotic resistance genes. Our research suggests that the presence of Tn6009 in the microbiome of the strain in combination with various antimicrobial-resistant genes may facilitate the transfer of resistance and virulence factors and subsequently contribute to the fitness and pathogenicity of *E. faecalis* IRMC827A. This could result in outbreaks in the future caused by other bacteria in the microbiome in addition to *E. faecalis*. In order to reduce outbreak conditions, additional molecular research is required to track the genomic and pathogenicity trends of clinical and carriage isolates across geographical areas. Antibiotic use should be considered carefully in both clinical and community settings in order to prevent the emergence and spread of antimicrobial resistance.

A recent study identified common and novel mutations in the *gyrA* and *parC* genes in *Pseudomonas* spp. clinical isolates from Saudi Arabia and provided insight into the genetic background of quinolone resistance [97]. A Saudi Arabian study found that the DNA gyrase in clinical isolates of *E. coli* targets quinolones, and that a single amino acid change in *gyrA* can make *E. coli* resistant to nalidixic acid and less sensitive to ciprofloxacin [98]. Another study on the qnr-positive isolates found various mutations in *gyrA*, but high-level ciprofloxacin resistance was linked to double mutations in *gyrA* among Enterobacteriaceae from Saudi Arabia [99]. Fluoroquinolone resistance was found to be linked to *gyrA* and *parC* gene mutations in *Salmonella enterica* from Riyadh, Saudi Arabia [100]. *E. faecalis* isolated from the western region of Saudi Arabia possessed *gyrA* and *parC* genes and exhibited resistance to quinolone antibiotics like ciprofloxacin, levofloxacin, and moxifloxacin, commonly used for UTIs, enteric infections, and respiratory tract infections [33]. These studies clearly indicate the presence of the *gyrA* gene in various bacterial isolates from the study country, which supports the resistance to antimicrobial agents as observed in the present study.

The international spread of multidrug-resistant microorganisms is a challenge facing healthcare providers today. It is crucial to gain some knowledge regarding local microorganisms in the country. The findings of this study clarified and shed light on the understanding of the molecular characteristics of *E. faecalis*. Nevertheless, the implementation of an active surveillance system to refine hospital protocols and policies, as well as introducing more legislation on the accessibility and usability of antimicrobial drugs, might be feasible strategies for now. Thus, future research must focus on developing new techniques for these infections, such as drugs that could target the resistance sites or rather an advanced treatment that acts on the resistance gene itself. Further studies are needed by either RNA sequencing or RT-PCR analysis of the most important genes to confirm their state of expression to ensure phenotype. Genotypes are the genetic makeup of an organism. WGS can be used to determine the genotype of an organism, but it cannot be used to directly determine the phenotype. The lack of phenotypic identification through antibiotic susceptibility tests from the isolate is one of the limitations of the study. Genetic makeup can be used to develop new diagnostic tests and treatments for antibiotic-resistant infections. It can also be used to track the spread of antibiotic resistance and to develop strategies to prevent it.

## 5. Conclusions

Many of the antibiotics commonly used to treat infections brought on by this pathogen, including *E. faecalis* IRMC827A, are highly resistant to it. The strain carries several virulence factors, including those that promote adherence to host cells, biofilm formation, and resistance to phagocytosis. Various mutations and mobile genetic elements (Tn6009 and ISS1N) in IRMC827A are associated with antibiotic resistance, which may contribute to the high level of resistance to antibiotics due to their ability to cause serious and fatal infections and be difficult to treat. The current observations of this study suggest that multidrug-resistant *E. faecalis* IRMC827A is a serious concern for public health. Further research is needed to develop novel antibiotics and effective treatment strategies for this pathogen.

## Figures and Tables

**Table 1:** Assembly details and annotated features of *Enterococcus faecalis* IRMC827A.

General Info	
Genome Name	*Enterococcus faecalis* IRMC827A
Taxonomy Info	
Superkingdom	Bacteria
Phylum	Firmicutes
Order	Lactobacillales
Family	Enterococcaceae
Genus	*Enterococcus*
Species	*Enterococcus faecalis*
Genome Statistics	
Contigs	42
Genome Length	2,899,764 bp
GC Content	37.34
Contig L50	6
Contig N50	193,505
Annotation Statistics	
CDS	2889
tRNA	41
Repeat Regions	7
rRNA	2
Hypothetical proteins	639
Proteins with functional assignments	2250
Proteins with EC number assignments	686
Proteins with GO assignments	564
Proteins with Pathway assignments	463
Proteins with genus-specific family (PLfam) assignments	2813
Proteins with cross-genus family (PGfam) assignments	2848

**Table 2:** Antimicrobial resistance genes and associated antimicrobial resistance mechanisms identified in the genome of *E. faecalis* IRMC827A.

S. No	Antimicrobial Resistant Mechanism	Name of the Genes
1	Antibiotic inactivation enzyme	*ANT(6)-I*, *CatA8 family*
2	Antibiotic target in susceptible species	*Alr*, *Ddl*, *EF-G*, *EF-Tu*, *folA*, *Dfr*, *folP*, *gyrA*, *gyrB*, *inhA*, *fabI*, *Iso-tRNA*, *kasA*, *MurA*, *rho*, *rpoB*, *rpoC*, *S10p*, *S12p*
3	Antibiotic target modifying enzyme	*RlmA(II)*
4	Antibiotic target protection protein	*Lsa(A)*, *Tet(M)*
5	Antibiotic target replacement protein	*FabK*
6	Efflux pump conferring antibiotic resistance	*Tet(L)*, *YkkCD*
7	Gene conferring resistance via absence	*gidB*
8	Protein altering cell wall charge conferring antibiotic resistance	*GdpD*, *MprF*, *PgsA*
9	Regulator modulating expression of antibiotic resistance genes	*LiaF*, *LiaR*, *LiaS*

**Table 3:** List of virulence factors in the genome of *E. faecalis* IRMC827A.

S. No	Source	Source ID	SO	Gene	Product	Classification	SC	QC	% Identity	E-Value
1	ResFinder 4.1/Victors	CP002491.1/29377514	b/c	*SrtA*	Sortase A, LPXTG specific		100	100	100	1 × 10^−134^
2	ResFinder 4.1	CP002491.1	b	*cCF10*					99.76	
3	ResFinder 4.1	295112306	e	*cOB1*					99.53	
4	ResFinder 4.1	CP002621.1	f	*cad*					99.89	
5	ResFinder 4.1	AF435439.1	g	*camE*	Sex pheromone cam373 precursor				99.80	
6	ResFinder 4.1/VFDB/Victors	CP003726.1/VFG042976/306753329	a/c/	*ebpA*	Von Willebrand factor type A domain protein	Adherence, Biofilm formation, Sortase-assembled pili	100	74	99	1 × 10^−130^
7	ResFinder 4.1	AE016830.1	c	*efaAfs*					99.68	
8	ResFinder 4.1	AE016830.1	c	*tpx*					99.61	
9	Victors	29376182	c	*EF1623*	Ethanolamine utilization protein similar to pdua/pduj		100	100	100	7 × 10^−46^
10	Victors/VFDB	29375537	c	*bopD*	Maltose operon transcriptional repressor malr, laci family	Biofilm formation	100	100	99	1 × 10^−190^
11	Victors	29375014	c	*EF0376*	Putative lipoprotein		100	100	100	1 × 10^−206^
12	VFDB	VFG002189	c	*cpsB*	Phosphatidate cytidylyltransferase	Antiphagocytosis	100	100	99	1 × 10^−147^
12	Victors	29376329	c	*purL*	Phosphoribosylformylglycinamidine synthase, synthetase subunit		100	100	99	0.0
14	Victors	67043736	m	*perR*	Peroxide stress regulator perr, FUR family		100	100	100	1 × 10^−82^
15	Victors	29376108	c	*recQ-1*	ATP-dependent DNA helicase recq		99	99	99	1 × 10^−275^
16	Victors	29376080	c	*EF1513*	ABC transporter, substrate-binding protein (cluster 5, nickel/peptides/opines)		100	100	99	0.0
17	Victors	29375449	c	*EF0861*	Acetyltransferase, GNAT family		100	100	99	1 × 10^−88^
18	Victors	29376708	c	*map*	Methionine aminopeptidase		100	100	100	1 × 10^−151^
19	Victors	29374885	c	*brnQ*	Na (+)-dependent branched-chain amino acid transporter		100	100	100	1 × 10^−256^
20	Victors	29376132	c	*psr*	Cell envelope-associated transcriptional attenuator lytr-cpsa-Psr, subfamily A1		100	100	99	1 × 10^−225^
21	VFDB	VFG002190	c	*cpsA*	Undecaprenyl diphosphate synthase	Antiphagocytosis	100	100	99	1 × 10^−156^
22	Victors	29375019	c	*EF0382*	Regulator of polyketide synthase expression		100	100	100	1 × 10^−304^
23	Victors	29377421	c	*EF2957*	Maltose O-acetyltransferase		100	100	100	1 × 10^−106^
24	VFDB	VFG002165	c	*efaA*	Manganese ABC transporter, periplasmic-binding protein sita	Adherence	100	100	99	1 × 10^−178^
25	Victors	29376164	c	*scrR-1*	Sucrose operon repressor scrr, laci family		100	99	100	1 × 10^−183^
26	Victors	29376105	c	*EF1542*	Hypothetical protein		71	100	99	1 × 10^−195^
27	Victors	29375870	c	*EF1302*	Transcriptional regulator		100	100	99	1 × 10^−167^
28	Victors	29377084	c	*EF2598*	PTS system, beta-glucoside-specific IIB component/PTS system, beta-glucoside-specific IIC component/PTS system, beta-glucoside-specific IIA component		100	100	99	0.0
29	Victors	29377078	c	*EF2591*	Glyoxalase family protein		100	97	100	1 × 10^−156^
30	VFDB	VFG042978	c	*ebpC*	Cell wall surface anchor family protein	Adherence, Biofilm formation, Sortase-assembled pili	100	100	99	0.0
31	VFDB/Victors	VFG042979/29375670	c	*srtC*	Sortase A, LPXTG specific	Adherence, Biofilm formation, Sortase-assembled pili	99	98	99	1 × 10^−160^
32	Victors	29375331	c	*EF0737*	Hypothetical protein		100	100	99	1 × 10^−298^
33	Victors	29376151	c	*EF1590*	N1-spermidine/spermine acetyltransferase paia		100	100	100	1 × 10^−100^
34	Victors	29376139	c	*thyA*	Thymidylate synthase		100	100	99	1 × 10^−189^

SO: Source organism; a = *E. faecalis* D32; b = *E. faecalis* 62; c = *E. faecalis* V583; e: *Enterococcus* sp. 7L76; f: *E. faecalis* OG1RF; g: *E. faecalis* strain OG1X; h: *Neisseria meningitidis* MC58; m: *E. faecalis*; SC: Subject coverage; QC: Query coverage. Complete list of virulence factors in the genome of *E. faecalis* IRMC827A presented in Table S1.

**Table 4:** List of resistance-phenotype-associated mutations in the genome of *E. faecalis* IRMC827A.

S. No	Gene	Mutation	Nucleotide Change	Amino Acid Change	Resistance Phenotype
1	*gyrA*	*gyrA* p.S83Y	AGT -> TAT	S -> Y	Nalidixic acid, Ciprofloxacin
2	*gyrA*	*gyrA* p.D759N	GAT -> AAT	D -> N	Unknown
3	*parC*	*parC* p.S80I	AGC -> ATC	S -> I	Nalidixic acid, Ciprofloxacin

## Data Availability

All data will be available on reasonable request from the corresponding author.

## Supplementary Materials

The following supporting information can be downloaded at: https://www.mdpi.com/article/10.3390/biology12101296/s1, Table S1: List of virulence factors in the genome of *E. faecalis* IRMC827A. Table S2: Metagenomic read mapping of *E. faecalis* IRMC827A through various databases. Table S3: Antimicrobial-resistant phenotype results observed in *E. faecalis* IRMC827A. Table S4: Antimicrobial-resistant phenotype specific for *Enterococcus faecalis* results observed in *E. faecalis* IRMC827A. Table S5: List of pathogenic protein families of the IRMC827A. Table S6: List of mobile genetic elements associated with antibiotic resistance of the IRMC827A. Figure S1: Multiple sequence alignment of dfrG gene from *Enterococcus faecalis* IRMC827A.
