# Supplementary material for: Genomic Landscape of Multidrug Resistance and Virulence in Enterococcus faecalis IRMC827A from a Long-Term Patient
Source: Biology (Basel). 2023 Sep 29;12(10):1296. doi: 10.3390/biology12101296 (PMC10604365; doi:10.3390/biology12101296)
Supplement: Supplementary file 1 [file biology-12-01296-s001.zip › biology-2585387-supplementary.pdf]

# Genomic Landscape of Multidrug Resistance and Virulence in *Enterococcus faecalis* IRMC827A from a Long-Term Patient

J. Francis Borgio [1], Reem AlJindan [2]*, Lujeen H Alghourab [3], Rahaf Alquwaie [4], Razan Aldahhan [1], Norah F Alhur[1], Doaa M. AlEraky [5], Nehal Mahmoud [2], Noor B. Almandil [6], Sayed AbdulAzeez [1*]

[1] Department of Genetic Research, Institute for Research and Medical Consultations (IRMC), Imam Abdulrahman Bin Faisal University, Dammam 31441, Saudi Arabia. Email: fbalexander@iau.edu.sa (JFB); raldahhan@iau.edu.sa (RazanA); norah.f.s.2@gmail.com (NFA); asayed@iau.edu.sa (SA)
[2] Department of Microbiology, College of Medicine, Imam Abdulrahman Bin Faisal University, Dammam 31441, Saudi Arabia. Email: raljindan@iau.edu.sa (RJ); nmhosin@iau.edu.sa (NM)
[3] Summer Research Program, Institute for Research and Medical Consultations (IRMC), Imam Abdulrahman Bin Faisal University, Dammam 31441, Saudi Arabia. Email: 2170004781@iau.edu.a (LHA)
[4] Master Program of Biotechnology, Institute for Research and Medical Consultations (IRMC), Imam Abdulrahman Bin Faisal University, Dammam 31441, Saudi Arabia. Email: 2230500195@iau.edu.sa (RahafA)
[5] Department of Biomedical Dental Science, Microbiology and Immunology Division, Collage of Dentistry, Dammam 31441, Saudi Arabia
[6] Department of Clinical Pharmacy Research, Institute for Research and Medical Consultations (IRMC), Imam Abdulrahman Bin Faisal University, Dammam 31441, Saudi Arabia. Email: nbalmandil@iau.edu.sa (NBA)

**Table S1.** List of virulence factors in the genome of *E. faecalis* IRMC827A.

| S. No | Source | Source ID | SO | Gene | Product | Classification | SC | QC | % Identity | E-Value |
|---|---|---|---|---|---|---|---|---|---|---|
| 1 | ResFinder 4.1 | CP003726.1 | a | *ElrA* | | | | | 98.76 | |
| 2 | ResFinder 4.1 / Victors | CP002491.1 / 29377514 | b / c | *SrtA* | Sortase A, LPXTG specific | | 100 | 100 | 100 | 1e-134 |
| 3 | ResFinder 4.1 / VFDB | AF260876.1 / VFG002166 | d / c | *ace* | Collagen adhesin | Adherence, MSCRAMM | 78 | 84 | 96 | 1e-296 |
| 4 | ResFinder 4.1 | CP002491.1 | b | *cCF10* | | | | | 99.76 | |
| 5 | ResFinder 4.1 | 295112306 | e | *cOB1* | | | | | 99.53 | |
| 6 | ResFinder 4.1 | CP002621.1 | f | *cad* | | | | | 99.89 | |
| 7 | ResFinder 4.1 | AF435439.1 | g | *camE* | Sex pheromone cam373 precursor | | | | 99.80 | |
| 8 | ResFinder 4.1 / VFDB / Victors | CP003726.1 / VFG042976 / 306753329 | a / c / | *ebpA* | Von Willebrand factor type A domain protein | Adherence, Biofilm formation, Sortase-assembled pili | 100 | 74 | 99 | 1e-130 |
| 9 | ResFinder 4.1 / VFDB | CP003726.1 / VFG042977 | a / C | *ebpB* | Cell wall surface anchor family protein | Adherence, Biofilm formation, Sortase-assembled pili | 100 | 100 | 98 | 1e-274 |
| 10 | ResFinder 4.1 | AE016830.1 | c | *efaAfs* | | | | | 99.68 | |
| 11 | ResFinder 4.1 | CP002491.1 | b | *hylA* | | | | | 98.93 | |
| 12 | ResFinder 4.1 | AE016830.1 | c | *tpx* | | | | | 99.61 | |
| 13 | Victors | 15676067 | h | *tufA* | Translation elongation factor Tu | | 98 | 98 | 81 | 1e-189 |
| 14 | Victors | 29376182 | c | *EF1623* | Ethanolamine utilization protein similar to pdua/pduj | | 100 | 100 | 100 | 7e-46 |
| 15 | Victors | 29376163 | c | *scrB-1* | Sucrose-6-phosphate hydrolase (EC | | 100 | 99 | 98 | 1e-293 |
| 16 | Victors / VFDB | 29375537 | c | *bopD* | Maltose operon transcriptional repressor malr, laci family | Biofilm formation | 100 | 100 | 99 | 1e-190 |
| 17 | VFDB | VFG043509 | c | *fss2* | Cell wall surface anchor family protein | | 25 | 100 | 95 | 1e-231 |
| 18 | Victors | 29375014 | c | *EF0376* | Putative lipoprotein | | 100 | 100 | 100 | 1e-206 |
| 19 | VFDB / Victors | VFG001359 / 15903537 | i / j | *psaA* | Manganese ABC transporter, periplasmic-binding protein sita | Manganese uptake, ABC transporter | 89 | 87 | 82 | 1e-135 |
| 20 | Patric / Victors | LMOh7858_1898 / 116516623 | k / l | *purB* | Adenylosuccinate lyase, SAICAR lyase | | 94 | 94 | 82 | 1e-200 |
| 21 | VFDB | VFG002189 | c | *cpsB* | Phosphatidate cytidylyltransferase | Antiphagocytosis | 100 | 100 | 99 | 1e-147 |
| 22 | Victors | 29377157 | c | *EF2675* | Competence protein coia | | 99 | 100 | 95 | 1e-226 |
| 23 | Victors | 29376329 | c | *purL* | Phosphoribosylformylglycinamidine synthase, | | 100 | 100 | 99 | 0.0 |

| | | | | | | | | | | |
|---|---|---|---|---|---|---|---|---|---|---|
| | | | | | synthetase subunit | | | | | |
| 24 | VFDB | VFG002196 | c | *EF3023* | Hyaluronate lyase precursor, polysaccharide lyase, family 8 | Exoenzyme, Spreading factor | 84 | 78 | 98 | 0.0 |
| 25 | VFDB | VFG043508 | c | *fss1* | Hypothetical protein | | 99 | 97 | 98 | 0.0 |
| 26 | Victors | 67043736 | m | *perR* | Peroxide stress regulator perr, FUR family | | 100 | 100 | 100 | 1e-82 |
| 27 | Victors | 29376108 | c | *recQ-1* | ATP-dependent DNA helicase recq | | 99 | 99 | 99 | 1e-275 |
| 28 | Victors | 29376080 | c | *EF1513* | ABC transporter, substrate-binding protein (cluster 5, nickel/peptides/opines) | | 100 | 100 | 99 | 0.0 |
| 29 | Victors | 29375449 | c | *EF0861* | Acetyltransferase, GNAT family | | 100 | 100 | 99 | 1e-88 |
| 30 | Victors / VFDB | 16804506 / VFG000077 | n | *clpP* | ATP-dependent Clp protease proteolytic subunit clpp | | 94 | 94 | 83 | 8e-87 |
| 31 | Victors | 29376708 | c | *map* | Methionine aminopeptidase | | 100 | 100 | 100 | 1e-151 |
| 32 | Victors | 29374885 | c | *brnQ* | Na (+)-dependent branched-chain amino acid transporter | | 100 | 100 | 100 | 1e-256 |
| 33 | Victors | 29376132 | c | *psr* | Cell envelope-associated transcriptional attenuator lytr-cpsa-Psr, subfamily A1 | | 100 | 100 | 99 | 1e-225 |
| 34 | VFDB | VFG002190 | c | *cpsA* | Undecaprenyl diphosphate synthase | Antiphagocytosis | 100 | 100 | 99 | 1e-156 |
| 35 | Victors | 29375019 | c | *EF0382* | Regulator of polyketide synthase expression | | 100 | 100 | 100 | 1e-304 |
| 36 | Victors | 29376338 | c | *EF1792* | Hypothetical protein | | 100 | 75 | 98 | 1e-39 |
| 37 | Victors | 29377421 | c | *EF2957* | Maltose O-acetyltransferase | | 100 | 100 | 100 | 1e-106 |
| 38 | Victors | 15901058 | i | *SP_1193* | Galactose-6-phosphate isomerase, laca subunit | | 99 | 99 | 83 | 1e-62 |
| 39 | VFDB / Victors | VFG002164 | c | *prgB/asc10* | Aggregation substance Asa1/prgb | Adherence | 56 | 100 | 97 | 0.0 |
| 40 | VFDB | VFG002165 | c | *efaA* | Manganese ABC transporter, periplasmic-binding protein sita | Adherence | 100 | 100 | 99 | 1e-178 |
| 41 | Victors | 29376164 | c | *scrR-1* | Sucrose operon repressor scrr, laci family | | 100 | 99 | 100 | 1e-183 |
| 42 | Victors | 29376159 | c | *phrB* | Deoxyribodipyrimidine photolyase | | 100 | 100 | 98 | 1e-286 |
| 43 | Victors | 29376105 | c | *EF1542* | Hypothetical protein | | 71 | 100 | 99 | 1e-195 |
| 44 | Victors | 29375870 | c | *EF1302* | Transcriptional regulator | | 100 | 100 | 99 | 1e-167 |
| 45 | Victors | 29377084 | c | *EF2598* | PTS system, beta-glucoside-specific IIB component / PTS system, beta-glucoside-specific IIC component / PTS system, beta-glucoside-specific IIA component | | 100 | 100 | 99 | 0.0 |
| 46 | Victors | 29377078 | c | *EF2591* | Glyoxalase family protein | | 100 | 97 | 100 | 1e-156 |
| 47 | VFDB | VFG042978 | c | *ebpC* | Cell wall surface anchor family protein | Adherence, Biofilm formation, Sortase-assembled pili | 100 | 100 | 99 | 0.0 |
| 48 | VFDB / Victors | VFG042979 / 29375670 | c | *srtC* | Sortase A, LPXTG specific | Adherence, Biofilm formation, Sortase- | 99 | 98 | 99 | 1e-160 |

| | | | | | | | | | | |
|---|---|---|---|---|---|---|---|---|---|---|
| | | | | | | assembled pili | | | | |
| 49 | Victors | 29375331 | c | *EF0737* | Hypothetical protein | | 100 | 100 | 99 | 1e-298 |
| 50 | Victors | 76788416 | o | *lepA* | Translation elongation factor lepa | | 99 | 98 | 83 | 1e-302 |
| 51 | Victors | 29376151 | c | *EF1590* | N1-spermidine/spermine acetyltransferase paia | | 100 | 100 | 100 | 1e-100 |
| 52 | Victors | 29376139 | c | *thyA* | Thymidylate synthase | | 100 | 100 | 99 | 1e-189 |

SO: Source organism; a = *E. faecalis* D32; b = *E. faecalis* 62; c = *E. faecalis* V583; d: *E. faecalis* strain MC02152/TX0024; e: *Enterococcus* sp. 7L76; f: *E. faecalis* OG1RF; g: *E. faecalis* strain OG1X; h: *Neisseria meningitidis* MC58; i: *Streptococcus pneumoniae* TIGR4; j: *S. pneumoniae* R6; k: *Listeria monocytogenes* serotype 4b str. H7858; l: *S. pneumoniae* D39; m: *E. faecalis*; n: *Listeria monocytogenes* EGD-e; o: *Streptococcus agalactiae* A909; SC: Subject coverage; QC: Query coverage.

**Table S2: Metagenomic read mapping of *E. faecalis* IRMC827A through various database.**

| Template | Function | Genome | GAS | EAS | TL | TI | TC | QI | QC | q value | p value |
|---|---|---|---|---|---|---|---|---|---|---|---|
| CARD\|BAE15963.1 | *Dfrg* | a | 501 | 3 | 498 | 100.00 | 100.00 | 100.00 | 100.00 | 489.79 | 1.0e-26 |
| CARD\|NZ_CP018138.1_1692408_1695312 | *S. pneumoniae* 23S rrna mutation conferring resistance to macrolides and streptogramins antibiotics | b | 1892 | 20 | 2904 | 89.29 | 100.38 | 88.95 | 99.62 | 1831.46 | 1.0e-26 |
| CARD\|CDO61513.1 | *Efra* | c | 1394 | 10 | 1457 | 98.63 | 99.59 | 99.04 | 100.41 | 1362.68 | 1.0e-26 |
| CARD\|AAO82601.1 | *E. faecalis* liaf mutant conferring daptomycin resistance | d | 711 | 5 | 732 | 98.91 | 100.00 | 98.91 | 100.00 | 694.70 | 1.0e-26 |
| CARD\|AEA93051.1 | *E. faecalis* cls conferring resistance to daptomycin | e | 1422 | 10 | 1446 | 99.38 | 100.00 | 99.38 | 100.00 | 1390.96 | 1.0e-26 |
| CARD\|NP_863168.1 | *E. faecium* chloramphenicol acetyltransferase | c | 528 | 4 | 648 | 93.67 | 100.00 | 93.67 | 100.00 | 513.47 | 1.0e-26 |
| CARD\|EOD99669.1 | *Dfre* | f | 459 | 3 | 495 | 97.37 | 100.00 | 97.37 | 100.00 | 447.85 | 1.0e-26 |
| CARD\|AAO82600.1 | *E. faecalis* lias mutant conferring daptomycin resistance | d | 1083 | 8 | 1104 | 99.28 | 100.00 | 99.28 | 100.00 | 1058.88 | 1.0e-26 |
| CARD\|CAJ67339.1 | *Tetm* | g | 1617 | 13 | 1920 | 94.69 | 100.00 | 94.69 | 100.00 | 1576.26 | 1.0e-26 |
| CARD\|YP_006374661.1 | *E. faecium* EF-Tu mutants conferring resistance to GE2270A | h | 882 | 9 | 1272 | 85.93 | 99.29 | 86.54 | 100.71 | 854.04 | 1.0e-26 |
| CARD\|AAA22851.1 | *Tet(L)* | i | 1371 | 9 | 1377 | 99.78 | 100.00 | 99.78 | 100.00 | 1341.36 | 1.0e-26 |
| CARD\|AAT46077.1 | *Lsaa* | j | 1443 | 10 | 1497 | 98.73 | 100.00 | 98.73 | 100.00 | 1410.90 | 1.0e-26 |
| CARD\|WP_002389492.1 | *E. faecalis* gshf with mutation conferring daptomycin resistance | k | 2232 | 15 | 2271 | 99.38 | 100.00 | 99.38 | 100.00 | 2185.32 | 1.0e-26 |
| CARD\|NP_816529.1 | *E. faecalis* liar mutant conferring daptomycin resistance | d | 621 | 4 | 633 | 99.21 | 100.00 | 99.21 | 100.00 | 606.84 | 1.0e-26 |
| VFDB\|VFG002165 | Endocarditis specific antigen | d | 921 | 5 | 927 | 99.68 | 100.00 | 99.68 | 100.00 | 904.26 | 1.0e-26 |
| VFDB\|VFG002166 | Collagen adhesin protein | d | 1644 | 11 | 2025 | 91.70 | 93.04 | 98.57 | 107.48 | 1608.56 | 1.0e-26 |
| VFDB\|VFG002189 | Phosphatidate cytidylyltransferase | d | 786 | 4 | 801 | 99.25 | 100.00 | 99.25 | 100.00 | 771.45 | 1.0e-26 |
| VFDB\|VFG002190 | Undecaprenyl diphosphate synthase | d | 798 | 4 | 816 | 99.14 | 100.00 | 99.14 | 100.00 | 783.19 | 1.0e-26 |
| VFDB\|VFG002196 | Polysaccharide lyase, family 8 | d | 3597 | 22 | 4119 | 98.37 | 106.99 | 91.94 | 93.46 | 3530.76 | 1.0e-26 |
| VFDB\|VFG002197 | Sugar-binding transcriptional regulator, laci family | d | 987 | 6 | 1011 | 99.11 | 100.00 | 99.11 | 100.00 | 968.79 | 1.0e-26 |
| VFDB\|VFG042976 | Endocarditis and biofilm-associated pilus tip protein *ebpa* | d | 3261 | 18 | 3312 | 99.46 | 100.00 | 99.46 | 100.00 | 3206.87 | 1.0e-26 |
| VFDB\|VFG042977 | Endocarditis and biofilm-associated pilus minor subunit *ebpb* | d | 1407 | 8 | 1431 | 99.37 | 100.00 | 99.37 | 100.00 | 1381.67 | 1.0e-26 |
| VFDB\|VFG042978 | Endocarditis and biofilm-associated pilus major subunit *ebpc* | d | 1855 | 10 | 1878 | 99.68 | 100.32 | 99.36 | 99.68 | 1822.37 | 1.0e-26 |
| VFDB\|VFG042979 | Sortase | d | 832 | 5 | 855 | 99.18 | 100.35 | 98.83 | 99.65 | 816.50 | 1.0e-26 |

| | | | | | | | | | | | |
|---|---|---|---|---|---|---|---|---|---|---|---|
| VFDB\|VFG043508 | *E. faecalis* surface protein Fss1, fibrinogen binding protein | d | 5700 | 29 | 5964 | 98.51 | 100.00 | 98.51 | 100.00 | 5613.26 | 1.0e-26 |

Template: Reference gene sequence; Genome: Genome that contains template gene; a: *Staphylococcus aureus*; b: *Streptococcus pneumoniae*; c: *E. faecium*; d: *E. faecalis* V583; e: *E. faecalis* OG1RF; f: *E. faecalis* EnGen0074; g: *Clostridium difficile* 630; h: *E. faecium* DO; i: *Geobacillus stearothermophilus*; j: *E. faecalis*; k: *Enterococcus.* GAS: Global alignment score of the template; EAS: Expected alignment score; TL: Template gene length in nucleotides; TI: Template Identity in percentage between the template and query, over the length of the matching query; TC: Template coverage in percentage of the template that is covered by the query; QI: Query identity in percentage between the query and template sequence, over the length of the matching query sequence; QC: Query coverage about the length of the matching query divided by the length of template; q value: Quantile from McNemars test, to test whether the current template is a significant hit.; p value: p-value corresponding to the obtained q-value.

**Table S3:** Antimicrobial resistant phenotype results observed in *Enterococcus faecalis* IRMC827A.

| # Antimicrobial | Class | WGS-predicted phenotype | Match | Genetic background |
|---|---|---|---|---|
| Tetracycline | Tetracycline | Resistant | 3 | *tet(L) (tet(L)_M29725), tet(M) (tet(M)_EU182585)* |
| Tigecycline | Tetracycline | No resistance | 0 | |
| Doxycycline | Tetracycline | Resistant | 3 | *tet(L) (tet(L)_M29725), tet(M) (tet(M)_EU182585)* |
| Minocycline | Tetracycline | Resistant | 2 | *tet(M) (tet(M)_EU182585)* |
| Metronidazole | Nitroimidazole | No resistance | 0 | |
| Rifampicin | Rifamycin | No resistance | 0 | |
| Hydrogen peroxide | Peroxide | No resistance | 0 | |
| Cefoxitin | Beta-lactam | No resistance | 0 | |
| Ampicillin | Beta-lactam | No resistance | 0 | |
| Cefepime | Beta-lactam | No resistance | 0 | |
| Ticarcillin | Beta-lactam | No resistance | 0 | |
| Amoxicillin | Beta-lactam | No resistance | 0 | |
| Ceftazidime | Beta-lactam | No resistance | 0 | |
| Ticarcillin+clavulanic acid | Beta-lactam | No resistance | 0 | |
| Ceftriaxone | Beta-lactam | No resistance | 0 | |
| Meropenem | Beta-lactam | No resistance | 0 | |
| Cephalotin | Beta-lactam | No resistance | 0 | |
| Unknown beta-lactam | Beta-lactam | No resistance | 0 | |
| Aztreonam | Beta-lactam | No resistance | 0 | |
| Piperacillin+tazobactam | Beta-lactam | No resistance | 0 | |
| Cephalothin | Beta-lactam | No resistance | 0 | |
| Cefixime | Beta-lactam | No resistance | 0 | |

| | | | | |
|---|---|---|---|---|
| Cefotaxime+clavulanic acid | Beta-lactam | No resistance | 0 | |
| Penicillin | Beta-lactam | No resistance | 0 | |
| Imipenem | Beta-lactam | No resistance | 0 | |
| Ampicillin+clavulanic acid | Beta-lactam | No resistance | 0 | |
| Ceftazidime+avibactam | Beta-lactam | No resistance | 0 | |
| Ertapenem | Beta-lactam | No resistance | 0 | |
| Piperacillin | Beta-lactam | No resistance | 0 | |
| Amoxicillin+clavulanic acid | Beta-lactam | No resistance | 0 | |
| Temocillin | Beta-lactam | No resistance | 0 | |
| Piperacillin+clavulanic acid | Beta-lactam | No resistance | 0 | |
| Cefotaxime | Beta-lactam | No resistance | 0 | |
| Quinupristin+dalfopristin | Streptogramin a | No resistance | 0 | |
| Dalfopristin | Streptogramin a | Resistant | 2 | *lsa(A) (lsa(A)_AY737526)* |
| Virginiamycin m | Streptogramin a | Resistant | 2 | *lsa(A) (lsa(A)_AY737526)* |
| Pristinamycin iia | Streptogramin a | Resistant | 2 | *lsa(A) (lsa(A)_AY737526)* |
| Temperature | Heat | No resistance | 0 | |
| Fosfomycin | Fosfomycin | No resistance | 0 | |
| Fusidic acid | Steroid antibacterial | No resistance | 0 | |
| Chloramphenicol | Amphenicol | Resistant | 2 | *cat (cat_U35036)* |
| Florfenicol | Amphenicol | No resistance | 0 | |
| Mupirocin | Pseudomonic acid | No resistance | 0 | |
| Linezolid | Oxazolidinone | No resistance | 0 | |
| Tiamulin | Pleuromutilin | No resistance | 0 | |
| Formaldehyde | Aldehyde | No resistance | 0 | |
| Ceftiofur | Under_development | No resistance | 0 | |
| Spectinomycin | Aminocyclitol | No resistance | 0 | |
| Butirosin | Aminoglycoside | No resistance | 0 | |
| Isepamicin | Aminoglycoside | No resistance | 0 | |
| Arbekacin | Aminoglycoside | No resistance | 0 | |
| Kasugamycin | Aminoglycoside | No resistance | 0 | |

| | | | | |
|---|---|---|---|---|
| Kanamycin | Aminoglycoside | No resistance | 0 | |
| Netilmicin | Aminoglycoside | No resistance | 0 | |
| Paromomycin | Aminoglycoside | No resistance | 0 | |
| Apramycin | Aminoglycoside | No resistance | 0 | |
| Unknown aminoglycoside | Aminoglycoside | No resistance | 0 | |
| Butiromycin | Aminoglycoside | No resistance | 0 | |
| Amikacin | Aminoglycoside | No resistance | 0 | |
| Astromicin | Aminoglycoside | No resistance | 0 | |
| Tobramycin | Aminoglycoside | No resistance | 0 | |
| Ribostamycin | Aminoglycoside | No resistance | 0 | |
| Neomycin | Aminoglycoside | No resistance | 0 | |
| Hygromycin | Aminoglycoside | No resistance | 0 | |
| Fortimicin | Aminoglycoside | No resistance | 0 | |
| Dibekacin | Aminoglycoside | No resistance | 0 | |
| Sisomicin | Aminoglycoside | No resistance | 0 | |
| Gentamicin | Aminoglycoside | No resistance | 0 | |
| Streptomycin | Aminoglycoside | Resistant | 3 | *str (str_X92946)* |
| Bleomycin | Aminoglycoside | No resistance | 0 | |
| Lividomycin | Aminoglycoside | No resistance | 0 | |
| Clindamycin | Lincosamide | Resistant | 2 | *lsa(A) (lsa(A)_AY737526)* |
| Lincomycin | Lincosamide | Resistant | 2 | *lsa(A) (lsa(A)_AY737526)* |
| Colistin | Polymyxin | No resistance | 0 | |
| Trimethoprim | Folate pathway antagonist | Resistant | 3 | *dfrG (dfrG_AB205645)* |
| Sulfamethoxazole | Folate pathway antagonist | No resistance | 0 | |
| Chlorhexidine | Quaternary ammonium compound | No resistance | 0 | |
| Benzylkonium chloride | Quaternary ammonium compound | No resistance | 0 | |
| Cetylpyridinium chloride | Quaternary ammonium compound | No resistance | 0 | |

| | | | | |
|---|---|---|---|---|
| Ethidium bromide | Quaternary ammonium compound | No resistance | 0 | |
| Carbomycin | Macrolide | No resistance | 0 | |
| Azithromycin | Macrolide | No resistance | 0 | |
| Telithromycin | Macrolide | No resistance | 0 | |
| Erythromycin | Macrolide | No resistance | 0 | |
| Oleandomycin | Macrolide | No resistance | 0 | |
| Spiramycin | Macrolide | No resistance | 0 | |
| Tylosin | Macrolide | No resistance | 0 | |
| Virginiamycin s | Streptogramin b | No resistance | 0 | |
| Pristinamycin ia | Streptogramin b | No resistance | 0 | |
| Quinupristin | Streptogramin b | No resistance | 0 | |
| Vancomycin | Glycopeptide | No resistance | 0 | |
| Teicoplanin | Glycopeptide | No resistance | 0 | |
| Fluoroquinolone | Quinolone | No resistance | 0 | |
| Unknown quinolone | Quinolone | No resistance | 0 | |
| Nalidixic acid | Quinolone | Resistant | 3 | *gyrA (p.S83Y), parC (p.S80I)* |
| Ciprofloxacin | Quinolone | Resistant | 3 | *gyrA (p.S83Y), parC (p.S80I)* |

Match: 0: No match found; 1: Match < 100% ID AND match length < ref length; 2: Match = 100% ID AND match length < ref length; 3: Match = 100% ID AND match length = ref length.

**Table S4:** Antimicrobial resistant phenotype specific for *Enterococcus faecalis* results observed in *E. faecalis* IRMC827A.

| # Antimicrobial | Class | WGS-predicted phenotype | Match | Genetic background |
|---|---|---|---|---|
| Tetracycline | Tetracycline | Resistant | 3 | *tet(L) (tet(L)_M29725), tet(M) (tet(M)_EU182585)* |
| Tigecycline | Tetracycline | No resistance | 0 | |
| Teicoplanin | Glycopeptide | No resistance | 0 | |
| Ciprofloxacin | Quinolone | Resistant | 3 | *gyrA (p.S83Y), parC (p.S80I)* |
| Erythromycin | Macrolide | No resistance | 0 | |
| Vancomycin | Glycopeptide | No resistance | 0 | |
| Linezolid | Oxazolidinone | No resistance | 0 | |
| Fosfomycin | Fosfomycin | No resistance | 0 | |
| Chloramphenicol | Amphenicol | Resistant | 2 | *cat (cat_U35036)* |
| Gentamicin | Aminoglycoside | No resistance | 0 | |

Match: 0: No match found; 1: Match < 100% ID AND match length < ref length; 2: Match = 100% ID AND match length < ref length; 3: Match = 100% ID AND match length = ref length.

**Table S5: List of pathogenic protein families of the IRMC827A.**

| | Organism | Class | Protein function | Protein ID | Identity |
|---|---|---|---|---|---|
| **1** | *Enterococcus faecalis* V583 | Lactobacillales | sigma-54 interaction domain protein | AAO80815 | 100% |
| **2** | *Enterococcus faecalis* V583 | Lactobacillales | lipoprotein, putative | AAO79950 | 99.61% |
| **3** | *Enterococcus faecalis* V583 | Lactobacillales | pheromone binding protein, putative | AAO79943 | 100% |
| **4** | *Enterococcus faecalis* V583 | Lactobacillales | peptide ABC transporter, peptide-binding protein | AAO80715 | 99.82% |
| **5** | *Enterococcus faecalis* V583 | Lactobacillales | pheromone binding protein | AAO82723 | 100% |
| **6** | *Enterococcus faecalis* V583 | Lactobacillales | membrane protein, putative | AAO81879 | 99.6% |
| **7** | *Enterococcus faecalis* V583 | Lactobacillales | DNA-binding response regulator, AraC family | AAO81949 | 99.59% |
| **8** | *Enterococcus faecalis* V583 | Lactobacillales | C4-dicarboxylate transporter, putative | AAO79983 | 100% |
| **9** | *Enterococcus faecalis* V583 | Lactobacillales | D-alanyl-D-alanine carboxypeptidase | AAO82807 | 99.59% |
| **10** | *Enterococcus faecalis* V583 | Lactobacillales | ribonuclease PH/Ham1 protein | AAO80922 | 99.75% |
| **11** | *Enterococcus faecalis* V583 | Lactobacillales | FolC family protein | AAO82616 | 100% |
| **12** | *Enterococcus faecalis* V583 | Lactobacillales | regulatory protein pfoR, putative | AAO79972 | 99.73% |
| **13** | *Enterococcus faecalis* V583 | Lactobacillales | transposase, putative | AAO80721 | 99.72% |
| **14** | *Enterococcus faecalis* V583 | Lactobacillales | carbohydrate kinase, pfkB family | AAO80652 | 99.70% |
| **15** | *Enterococcus faecalis* V583 | Lactobacillales | sugar-binding transcriptional regulator, | AAO80046 | 100% |
| **16** | *Enterococcus faecalis* V583 | Lactobacillales | iron compound ABC transporter, iron compound-binding protein | AAO81420 | 100% |
| **17** | *Enterococcus faecalis* V583 | Lactobacillales | aminoglycoside 6-adenylyltransferase | AAO82553 | 99.65% |
| **18** | *Enterococcus faecalis* V583 | Lactobacillales | MutT/nudix family protein | AAO82404 | 100% |
| **19** | *Enterococcus faecalis* V583 | Lactobacillales | PTS system, IIC component | AAO82884 | 100% |
| **20** | *Enterococcus faecalis* V583 | Lactobacillales | conserved hypothetical protein | AAO80309 | 99.61% |

| | | | | | |
|---|---|---|---|---|---|
| **21** | *Enterococcus faecalis* V583 | Lactobacillales | phosphosugar-binding transcriptional regulator, RpiR family, putative | AAO82731 | 100% |
| **22** | *Enterococcus faecalis* V583 | Lactobacillales | hydrolase, haloacid dehalogenase-like family | AAO82386 | 99.58% |
| **23** | *Enterococcus faecalis* V583 | Lactobacillales | cell division protein DivIVA | AAO80808 | 100% |
| **24** | *Enterococcus faecalis* V583 | Lactobacillales | MgtC family protein | AAO80322 | 100% |
| **25** | *Enterococcus faecalis* V583 | Lactobacillales | transcriptional regulator, Crp/Fnr family | AAO79952 | 100% |
| **26** | *Enterococcus faecalis* V583 | Lactobacillales | nitroreductase family protein, putative | AAO80478 | 100% |
| **27** | *Enterococcus faecalis* V583 | Lactobacillales | conserved hypothetical protein | AAO82554 | 100% |
| **28** | *Enterococcus faecalis* V583 | Lactobacillales | conserved hypothetical protein | AAO82735 | 100% |
| **29** | *Enterococcus faecalis* V583 | Lactobacillales | transcriptional regulator, TetR family | AAO82741 | 100% |
| **30** | *Enterococcus faecalis* V583 | Lactobacillales | conserved hypothetical protein | AAO81470 | 100% |
| **31** | *Enterococcus faecalis* V583 | Lactobacillales | signal peptidase I | AAO81456 | 100% |
| **32** | *Enterococcus faecalis* V583 | Lactobacillales | conserved hypothetical protein | AAO80229 | 100% |
| **33** | *Enterococcus faecalis* V583 | Lactobacillales | PTS system, IIB component | AAO82883 | 100% |
| **34** | *Enterococcus faecalis* V583 | Lactobacillales | conserved hypothetical protein | AAO82774 | 100% |
| **35** | *Enterococcus faecalis* V583 | Lactobacillales | transcriptional regulator SrlR | AAO82973 | 100% |
| **36** | *Enterococcus faecalis* V583 | Lactobacillales | PTS system, IIA component | AAO80516 | 100% |
| **37** | *Enterococcus faecalis* V583 | Lactobacillales | transcriptional regulator, MarR family | AAO81446 | 100% |
| **38** | *Enterococcus faecalis* V583 | Lactobacillales | PTS system, IIA component, putative | AAO82882 | 100% |
| **39** | *Enterococcus faecalis* V583 | Lactobacillales | PTS system, IIA component | AAO80316 | 100% |
| **40** | *Enterococcus faecalis* V583 | Lactobacillales | nrdI protein | AAO80327 | 100% |
| **41** | *Enterococcus faecalis* V583 | Lactobacillales | Conserved hypothetical protein | AAO81798 | 100% |
| **42** | *Enterococcus faecalis* V583 | Lactobacillales | Conserved hypothetical protein | AAO82991 | 100% |

| | | | | | |
|---|---|---|---|---|---|
| **43** | *Enterococcus faecalis* V583 | Lactobacillales | competence protein | AAO81779 | 100% |
| **44** | *Enterococcus faecalis* V583 | Lactobacillales | ferredoxin | AAO81330 | 100% |
| **45** | *Enterococcus faecalis* V583 | Lactobacillales | conserved hypothetical protein | AAO80806 | 100% |
| **46** | *Enterococcus faecalis* V583 | Lactobacillales | ribosomal protein L29 | AAO80083 | 100% |
| **47** | *Enterococcus faecalis* V583 | Lactobacillales | hypothetical protein | AAO81074 | 100% |
| **48** | *Enterococcus faecalis* V583 plasmid pTEF1 | Lactobacillales | conserved domain protein | AAO83066 | 100% |
| **49** | *Enterococcus faecalis* V583 plasmid pTEF1 | Lactobacillales | site-specific recombinase, resolvase family | AAO83065 | 100% |
| **50** | *Enterococcus faecalis* V583 plasmid pTEF1 | Lactobacillales | mpB/MucB/SamB family protein | AAO83069 | 99.77% |
| **51** | *Streptococcus pneumoniae* G54 | Lactobacillales | Tn5251 transcriptional regulator Cro/CI family | ACF55955 | 100% |
| **52** | *Staphylococcus aureus subsp. aureus* Mu3 DNA | Bacillales | plasmid recombination enzyme | BAF76914 | 100% |
| **53** | *Streptococcus suis* 05ZYH33 | Lactobacillales | Tn916, transposase | ABP89883 | 100% |
| **54** | *Streptococcus agalactiae* 2603V/R | Lactobacillales | Tn916, hypothetical protein | AAM99805 | 100% |
| **55** | *Streptococcus pneumoniae* Hungary19A-6 | Lactobacillales | conserved hypothetical protein | ACA36408 | 100% |

**Table S6: List of mobile genetic elements associated with antibiotic resistance of the IRMC827A.**

| MGE no | name | prediction method | type | allele_len | e_ value | identity | coverage | gaps | substitution | contig | start | stop | cigar |
|---|---|---|---|---|---|---|---|---|---|---|---|---|---|
| 1 | Tn6009 | alignment to reference | Integrative Conjugative Element | 1889 | 0 | 0.999471 | 1 | 0 | 1 | Scaffold2 | 31342 | 33230 | M1889 |
| 6 | ISS1N | alignment to reference | Insertion sequence | 808 | 0 | 0.985149 | 1 | 0 | 12 | Scaffold22 | 16162 | 16969 | M808 |

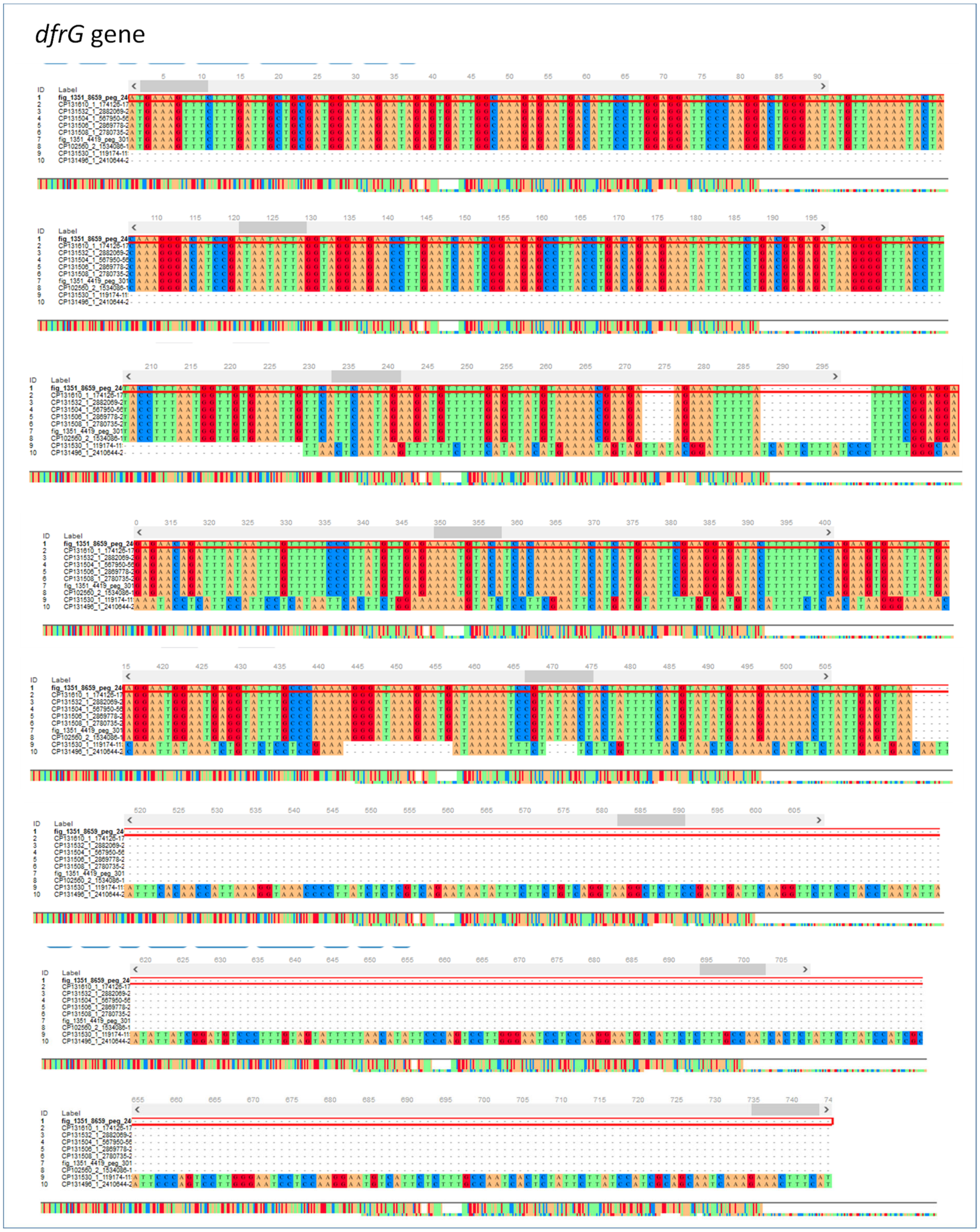


**Figure S1:** Multiple sequence alignment of *dfrG* gene from *Enterococcus faecalis* IRMC827A.
